# Clinical and molecular epidemiology of *Clostridioides difficile* infection in a county-level hospital in Zhejiang Province, China

**DOI:** 10.3389/fmicb.2026.1882254

**Published:** 2026-08-04

**Authors:** Meihong Wang, Liping Yu, Lisi Zheng, Xiajing Bi, Xiaobei Xu, Haiying Fang, Tao Lv, Yunbo Chen

**Affiliations:** 1 Department of Laboratory Medicine, Chun'an County hospital of Traditional Chinese Medicine, Hangzhou, China; 2 State Key Laboratory for Diagnosis and Treatment of Infectious Diseases, Collaborative Innovation Center for Diagnosis and Treatment of Infectious Diseases, The First Affiliated Hospital, Zhejiang University School of Medicine, Hangzhou, China; 3 Department of Nursing, The First Affiliated Hospital, Zhejiang University School of Medicine, Hangzhou, China

**Keywords:** antibiotic resistance, *Clostridioides difficile* infection, molecular epidemiology, transmission, whole genome sequencing

## Abstract

**Background:**

*Clostridioides difficile* infection (CDI) remains an important healthcare-associated infection worldwide. However, the molecular epidemiology of CDI in county-level hospitals across China remains poorly understood. Therefore, this study aimed to investigate the clinical and molecular epidemiology of CDI in a county-level hospital in Zhejiang Province, China.

**Methods:**

Between January and December 2025, we conducted a genomic epidemiology study of *C. difficile* isolates recovered from patients with clinically suspected CDI at a county-level hospital in Zhejiang Province, China. *C. difficile* strains were isolated and identified, typed by multi-locus sequence typing (MLST), and phylogenetic analysis, core-genome single-nucleotide polymorphism (cgSNP) analysis, and antimicrobial resistance (AMR) genes identification were extracted from whole genome sequencing. Antimicrobial susceptibility testing was performed using the agar dilution method according to CLSI guidelines. Statistical analysis was performed using R/Python.

**Results:**

In total, 50 (3.8%) toxigenic *C. difficile* isolates were identified from a total 1,315 stool samples. Among them, 40 (80.0%) exhibited the *tcdA*+/*tcdB*+/CDT– profile, 8 (16.0%) exhibited the *tcdA*–/*tcdB*+/CDT– profile, and 2 (4.0%) exhibited the *tcdA*+/*tcdB*+/CDT+ profile. And 16 (32.0%) strains were isolated from the Digestive department, followed by 8 (16.0%) strains from the Rehabilitation department. The most sequence type of the toxigenic *C. difficile* was ST2 (14.0%) and ST3 (14.0%), followed by ST81 (12.0%), ST35 (10.0%) and ST129 (8.0%). cgSNP-based minimum spanning tree analysis provided genomic evidence compatible with potential nosocomial transmission, including ST3, ST35, ST81, and ST129, with closely related isolates identified both within and between departments. Furthermore, all toxigenic isolates were successfully analyzed for antimicrobial susceptibility phenotype and remained susceptible to vancomycin, metronidazole, tigecycline, daptomycin, and fidaxomicin. High resistance rates were observed for clindamycin and erythromycin, while moderate resistance to tetracycline and moxifloxacin was also detected. Resistance phenotypes generally correlated with the presence of corresponding resistance determinants, including *ermB, tetM*, and the *gyrA* (Thr82Ile) mutation.

**Conclusions:**

This study provides insight into the molecular epidemiology and antimicrobial resistance characteristics of toxigenic *C. difficile* in a county-level hospital in China. Our findings show potential healthcare-associated transmission of several predominant lineages and highlight the importance of continuous genomic surveillance and strengthened infection prevention and control strategies to limit the dissemination of epidemic and multidrug-resistant *C. difficile* strains in healthcare settings.

## Introduction

*Clostridioides difficile* (*C. difficile*) is a Gram-positive, spore-forming anaerobic bacterium and a leading cause of healthcare-associated infection worldwide (Sandhu and McBride, 2018). Clinically, *C. difficile* infection (CDI) ranges from mild diarrhea to severe pseudomembranous colitis and can lead to substantial morbidity and mortality (Guh and Kutty, 2018). About 20–30% of the cases of antibiotic-associated diarrhea and 90% of pseudomembranous colitis are caused by *C. difficile* (Barbut and Petit, 2001; Hull and Beck, 2004). Over the past two decades, the incidence and severity of CDI have increased substantially, largely driven by the widespread use of broad-spectrum antibiotics and the emergence of hypervirulent strains. Currently, *C. difficile* infection still represents a major public health burden for global health-care systems (Rodriguez et al., 2016). Surveillance data from the United States reported an incidence of 116.1 cases per 100,000 population in 2022, while earlier estimates reached up to 130.28 cases per 100,00 persons (Rabiee et al., 2026; Zhang et al., 2016). Similarly, European countries have reported substantial CDI incidence, with rates varying between 58 and 138 cases per 100,000 population depending on the country and surveillance system (Brestrich et al., 2023; Nuttens et al., 2026). These findings highlight the importance of genomic epidemiological surveillance of *C. difficile* infection.

In China, the epidemiology of CDI remains less well defined. Available studies suggest that the overall incidence is lower than that reported in Western countries but may be increasing. A nationwide estimate reported a crude incidence of approximately 34 cases per 100,000 population during 2009–2016 (Liao et al., 2018). Previous epidemiological investigation and other hospital-based studies discovered that the CDI incidence rang from approximately 3.4 to 7 per 10,000 admissions (Xu et al., 2017). In addition, a meta-analysis showed that the prevalence of CDI patients with diarrhea is approximated 11.4% (Wen et al., 2023). Obviously, the overall incidence is lower than that in Western countries (Jin et al., 2017). This discrepancy may be attributable to differences in diagnostic awareness, testing capacity, and surveillance systems. Actually, these reported incidence rates likely represent only the tip of the iceberg, suggesting that the true burden of CDI in China is likely underestimated.

Currently, previous molecular epidemiological studies in China have demonstrated a strain distribution distinct from that observed in North America and Europe. Sequence types (STs) such as ST2, ST3, ST35, ST37, ST54, and ST81 are consistently reported as the predominant circulating lineages, whereas the epidemic RT027/ST1 and RT078/ST11 lineages remain uncommon (Liao et al., 2018; Xu et al., 2017; Lu et al., 2026). Meanwhile, molecular epidemiological studies of *C. difficile* in China have mainly been conducted in large tertiary hospitals, where diagnostic facilities and research capacity are more advanced. In contrast, CDI remains under-recognized in many county-level and smaller regional hospitals, and molecular epidemiological investigations are rarely performed in these settings. As a result, data on strain distribution, transmission dynamics, and antimicrobial resistance patterns are limited. To address this gap, the present study aimed to investigate the clinical and molecular epidemiology of CDI in a county-level hospital, thereby providing additional evidence to improve the understanding of CDI in underrepresented healthcare settings.

## Materials and methods

### Study design, population and *C. difficile* isolates

This study was conducted at the Chun'an County Hospital of Traditional Chinese Medicine, a county-level hospital in Hangzhou, China. Unformed stool samples were collected from inpatients and outpatients with clinically suspected CDI who presented with diarrhea between January 1, 2025 and December 30, 2025. During the cultivation process, anaerobic isolation of *C. difficile* was performed using the selective medium cycloserine-cefoxitin-taurocholate agar (CCFA-TA; Oxoid, UK). And the plates were incubated under anaerobic conditions for 48 h at 37°C. The suspected *C. difficile* colonies were identified using Bruker matrix-assisted laser desorption/ionization-time of flight mass spectrometry (Bruker Daltonik GmbH, Bremen, Germany) according to the operating manual. Bacteria grown on anaerobic blood agar for 48 h were suspended in 1 mL of distilled water, boiled for 15 min, and centrifuged (15,000 × g, 2 min). The supernatant was subsequently used for PCR detection of the *tcdA, tcdB*, and binary toxin genes, as previously described (Chen et al., 2014).

Clinical data were collected through the hospital's electronic medical record system, including the patient's gender, age, ward, and date of admission. But Diarrhea was defined as three loose stools within at least one 24 h period (van Prehn et al., 2021). A case of CDI was defined as diarrheal when the patient was positive for *C. difficile* culture and toxin tests (van Prehn et al., 2021).

### Whole genome sequencing, assembly and data analysis

The genomic DNA was extracted using the FastDNA^®^ Spin Kit for Soil (MP Biomedicals, Illkirch, France). Strains were subjected to whole genome sequencing, performed on the Illumina NovaSeq X Plus platform (Illumina, San Diego, CA, USA), achieving 200 × sequencing coverage. Before assembly, raw sequence reads were subjected to quality control using FastQC (https://www.bioinformatics.babraham.ac.uk/projects/fastqc/) and adapter regions trimmed using Trimmomatic (Bolger et al., 2014). Trimmed reads were assembled *de novo* using SPAdes with the default parameters, with a scaffold N50 length of approximately 0.5 Mb and a guanine-cytosine (GC) content of about 28.93% (Bankevich et al., 2012). Multi-locus sequence typing (MLST) was performed using the PubMLST sequence query page (https://pubmlst.org/). Genome annotations were performed on the RAST server (rast.nmpdr.org) and with Prokka (Seemann, 2014).

### Identification of antimicrobial resistance genes, virulence genes and transposons

Antibiotic resistance genes were identified using the Comprehensive Antibiotic Resistance Database (CARD) Resistance Gene Identifier (RGI) software (https://card.mcmaster.ca/analyze/rgi), and virulence genes including specific *C. difficile*-associated toxins, was predicted based on the Virulence Factors of Pathogenic Bacteria Database web server (http://www.mgc.ac.cn/VFs/). Transposons were identified using Vrprofile2 (Wang et al., 2022).

### Phylogenetic analysis and single nucleotide polymorphism analysis

The maximum likelihood trees based on core genome of all *C. difficile* strains were constructed using MEGA11 with 1000 bootstrap replicates and visualized using the Interactive Tree of Life (iTOL) web server (Letunic and Bork, 2011; Tamura et al., 2021). The chromosome of CD630 (AM180355.1) was set as the reference for toxigenic *C. difficile* strains in this study. Variant calls for single nucleotide polymorphisms (SNPs) were performed using Snippy (https://github.com/tseemann/snippy) with default parameters. The alignment file was filtered from variants with elevated densities of base substitutions as putative repetitive regions, mobile genetic elements and recombination events by Gubbins (Croucher et al., 2015), and used to calculate the pairwise core-genome single-nucleotide polymorphism (cgSNP). A threshold of ≤ 3 cgSNPs was established to identify strains possibly involved in transmission events (Eyre et al., 2013).

### Antimicrobial susceptibility testing

Antimicrobial susceptibility testing was performed using the agar dilution method according to guidelines of the Clinical and Laboratory Standards Institute (CLSI). *C. difficile* ATCC700057 was used as the quality control strain. The following 15 antimicrobial agents were tested: vancomycin, metronidazole, clindamycin, erythromycin, imipenem, moxifloxacin, tetracycline, linezolid, rifampin, tigecycline, fidaxomicin, ceftriaxone, chloramphenicol, daptomycin, and rifaximin. In addition to CLSI criteria, resistance breakpoints were determined based on the European Committee on Antimicrobial Susceptibility Testing (EUCAST) guidelines for antibiotics for which CLSI recommendations are not available, including vancomycin (>2 μg/mL), linezolid (>4 μg/mL) and rifampicin (>32 μg/mL) (http://www.eucast.org/clinical_breakpoints/).

### Statistical analysis

Categorical variables were compared using the chi-square test or Fisher's exact test, as appropriate. An exploratory multivariable logistic regression analysis was performed to evaluate factors associated with CDI. Variables were selected a priori based on clinical relevance and the availability of complete data for all enrolled patients. Because detailed clinical information was not consistently available, only age, sex, and hospital department were included in the final multivariable model. Odds ratios (ORs) and 95% confidence intervals (CIs) were calculated. A two-tailed *P* value < 0.05 was considered statistically significant. In view of the relatively limited number of CDI cases, the multivariable analysis was just exploratory in nature. Statistical analysis was performed using R/Python.

## Results

### Sample collection and *C. difficile* isolation in the hospital

Over the period of 12 months, a total of 1,315 non-repeated stool samples were collected. The age of patients in this study ranged between 1 and 101 years (median age: 61 years). Patients in neurology (mean age: 73.44 years), cardiology (mean age: 69.51 years), ICU (mean age: 67.00 years), and pneumology (mean age: 68.17 years) were predominantly elderly. Patients in the oncology (mean age: 65.32 year), orthopedics (mean age: 64.96 years), rehabilitation (mean age: 64.19 years) and digestive (mean age: 53.86 years) departments were also relatively older, while outpatient and emergency departments were characterized by younger populations (Supplementary Table 1).

**Figure 1:**
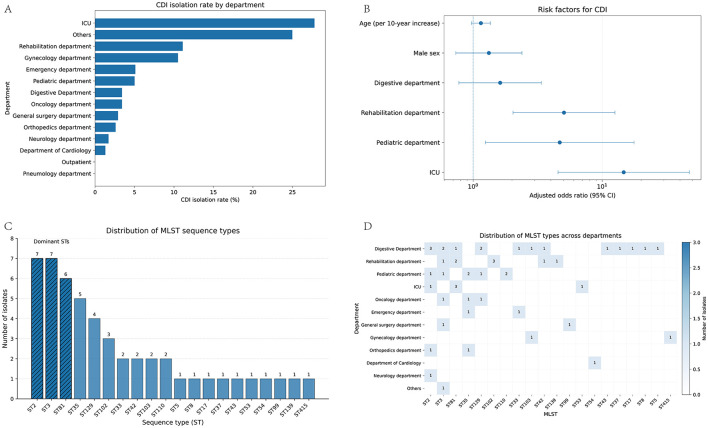
Molecular epidemiology of toxigenic *C. difficile* isolates. **(A)** isolation rate of toxigenic *C. difficile* by department. **(B)** Forest plot of factors associated with CDI. **(C)** Distribution of multi-locus sequence typing (MLST) sequence types. **(D)** Distribution of MLST sequence types across departments.

*C. difficile* was isolated from 64 stools, of which 50 were positive for toxigenic *C. difficile*. The total isolation rate of toxigenic *C. difficile* was 3.8%. Among the toxigenic isolates from patients, 40 (80.0%) exhibited the *tcdA*+/*tcdB*+/CDT– profile, 8 (16.0%) exhibited the *tcdA*–/*tcdB*+/CDT– profile, and 2 (4.0%) exhibited the *tcdA*+/*tcdB*+/CDT+ profile. And 16 (32.0%) strains were isolated from the Digestive department, followed by 8 (16.0%) strains from the Rehabilitation department, 7 (14.0%) strains from the Pediatric department, 5 (10.0%) strains from the intensive care unit (ICU) and 3 (6.0%) strains from Oncology department. Meanwhile, the isolation rate of toxigenic *C. difficile* from the Digestive department, Rehabilitation department, Pediatric department, ICU and Oncology department were 3.4% (16/474), 11.1% (8/72), 5.0% (7/140), 27.8% (5/18) and 3.4% (3/87), respectively (Figure 1A and Supplementary Table 2). The highest frequencies were observed in the ICU, rehabilitation department (Supplementary Table 2 and Figure 1D). The distribution of CDI cases differed significantly across departments (*P* < 0.001).

### Molecular epidemiology of the isolates

**Figure 2:**
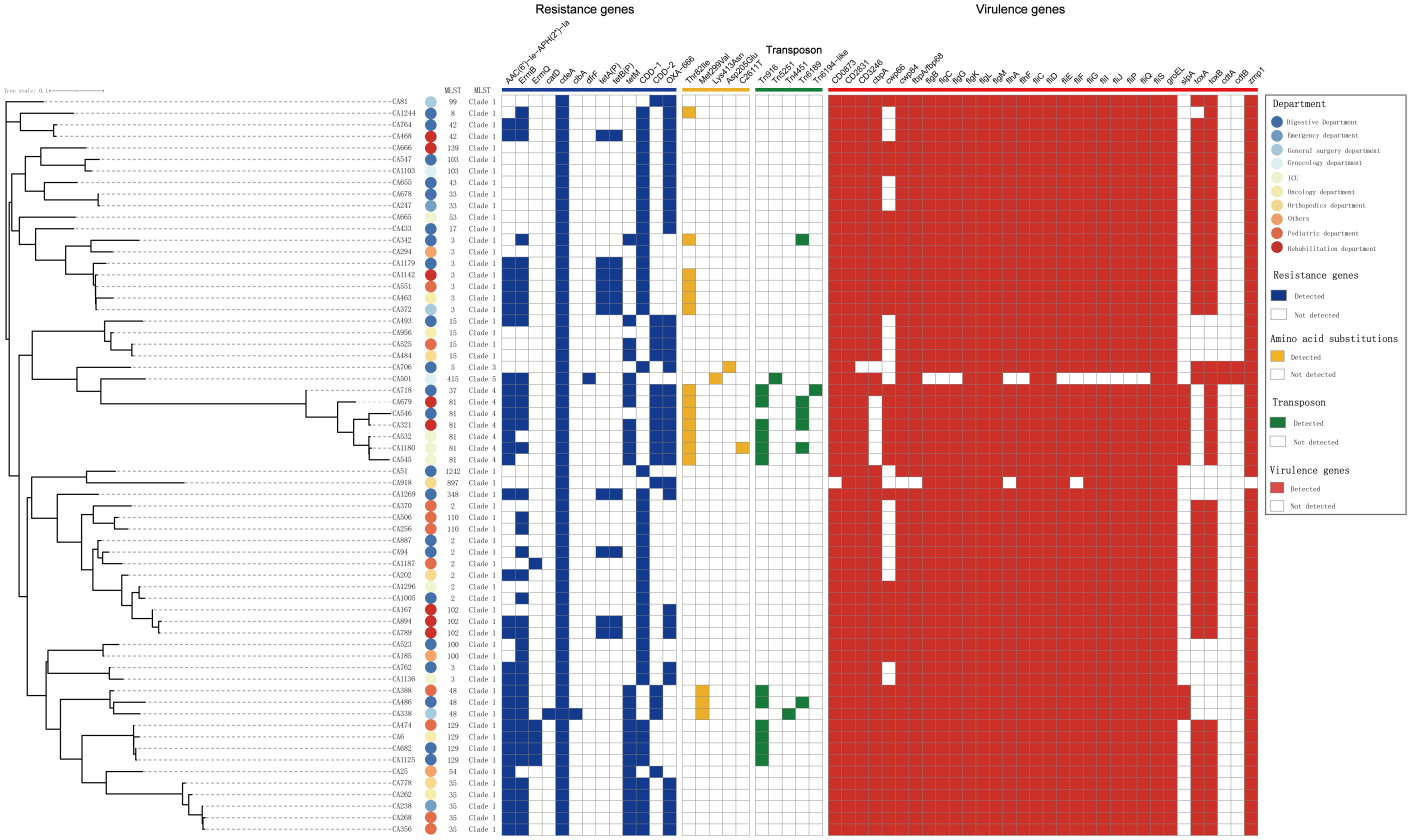
Maximum likelihood phylogenetic tree of 64 *C. difficile* strains was constructed based on the core genome using Roary. The annotations indicate the strain number, hospital departments, the multi-locus sequence typing (MLST), MLST clade, antimicrobial resistance gene, mobile genetic elements, resistance-associated amino acid substitutions, and virulence genes.

All *C. difficile* strains were analyzed by MLST and divided into 26 different STs, of which 20 were the toxigenic *C. difficile* (Figures 1C, 2). Based on the MLST clade classification, most isolates belonged to Clade 1. None of the isolates was identified as ST1 (BI/NAP1/027) or ST-11, which comprises the hypervirulent ribotypes RT078, RT126, and RT127. Notably, one isolate each belonged to ST5 (Clade 3) and ST415 (Clade 5), both of which carried *tcdA, tcdB*, and binary toxin genes. Among the toxigenic isolates, all ST8, ST37, and ST81 isolates exhibited only the TcdB gene. The most sequence type of the toxigenic *C. difficile* was ST2 (14.0%) and ST3 (14.0%), followed by ST81 (12.0%), ST35 (10.0%) and ST129 (8.0%). Among them, ST102 was predominantly identified in the rehabilitation department, ST2 was mainly detected in the digestive department, and ST81 was enriched in ICU settings. Several other STs, including ST3, ST35, ST110, and ST129, also showed non-random distributions across departments.

### Phylogenetic analysis and core-genome analysis

A phylogenetic tree was constructed based on the core-genome of all *C. difficile* strains. The phylogenetic tree revealed multiple evolutionary clusters that were broadly congruent with ST lineages and toxin gene profiles. Of note, ST3 strains separated into two clusters, a toxigenic cluster and non-toxigenic cluster.

**Figure 3:**
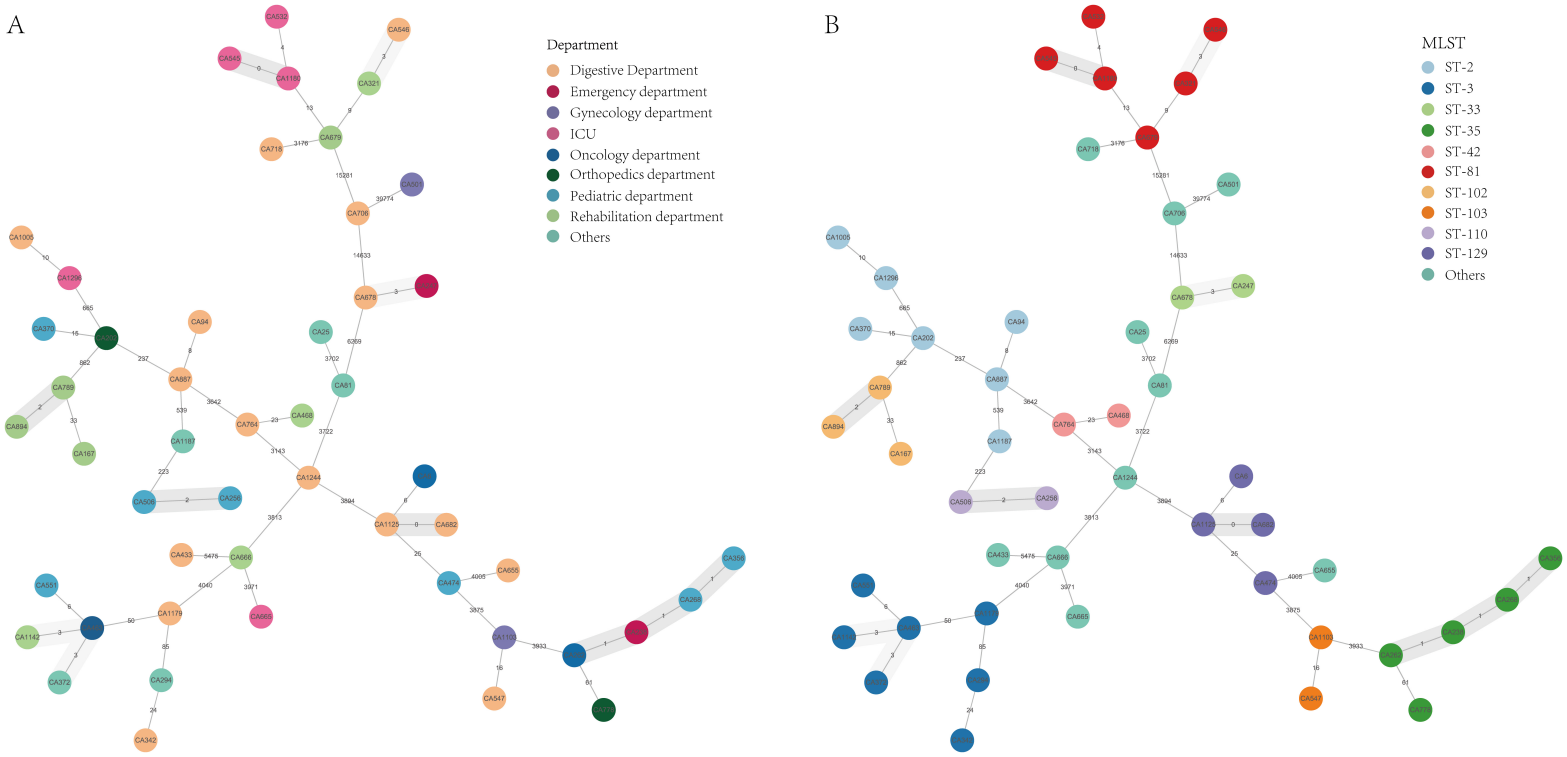
Minimum spanning trees of 50 toxigenic *C. difficile* strains based on the pairwise core-genome single-nucleotide polymorphism distances using Snippy. Related genotypes ( ≤ 3 alleles distance) are shaded in gray. **(A)** Circle colors indicate the hospital departments from which each isolate was obtained. **(B)** Circle colors indicate the MLST of each isolate.

To further investigate the genetic relationships and research the clonal transmission characteristics of these toxigenic *C. difficile* strains, a minimum spanning tree was constructed based on the core-genome single-nucleotide polymorphism (cgSNP) (Figure 3). Several toxigenic *C. difficile* isolates exhibited extremely close genetic relatedness. Toxigenic *C. difficile* isolates CA455 and CA1180 belonged to ST81 from ICU patients extremely close genetic relatedness with 0 cgSNP. Similarly, within the ST129 lineage, isolates CA1125 and CA682 from the digestive department differed by 0 cgSNPs. In the ST35 strains, isolates CA262 from the oncology department and CA238 from the emergency department differed by only 1 cgSNP. In addition, ST3 isolates from different patients also formed closely related clusters with minimal cgSNP differences.

### Comparison of antimicrobial resistance phenotypes and genotypes of toxigenic *C. difficile* strains

The distribution of minimum inhibitory concentrations of 15 antimicrobial agents against 50 toxigenic *C. difficile* strains is shown in Table 1. All isolates remained fully susceptible to vancomycin, metronidazole, tigecycline, daptomycin and fidaxomicin. In contrast, high resistance rates were observed for clindamycin (100.0%) and erythromycin (66.0%, 33/50). Moderate resistance rates were detected for tetracycline (38.0%, 19/50) and moxifloxacin (36.0%, 18/50), while lower resistance rates were observed for imipenem (24.0%, 12/50) and ceftriaxone (12.0%, 6/50). Linezolid, rifampin, rifaximin and chloramphenicol showed high activity with minimal resistance.

**Table 1:** MICs of the 15 antimicrobial agents against toxigenic *C. difficile* strains.

Antibiotic	S (%)	I (%)	R (%)	MIC_50_ (mg/L)	MIC_90_ (mg/L)	Range (mg/L)
Vancomycin	100	0	0	0.5	1	0.5–1
Metronidazole	100	0	0	0.25	0.5	0.125–0.5
Clindamycin	0	0	100	32	32	8–32
Erythromycin	34	0	66	128	256	1–256
Imipenem	14	62	24	8	16	2–64
Moxifloxacin	62	2	36	2	32	0.5–32
Tetracycline	46	16	38	8	32	0.06–64
Linezolid	96	0	4	1	2	1–8
Rifampin	96	2	2	0.06	0.06	0.06–128
Tigecycline	100	0	0	0.06	2	0.06–2
Fidaxomicin	100	0	0	0.06	0.25	0.015–0.5
Ceftriaxone	70	18	12	16	64	8–256
Chloramphenicol	90	8	2	8	8	2–32
Daptomycin	100	0	0	0.5	1	0.125–1
Rifaximin	96	0	4	0.008	0.008	0.008–64

MIC_50_ and MIC_90_ represent concentrations inhibiting 50% and 90% of isolates.

*C. difficile* presents resistance to different types of antibiotics due to the fact that its genome contains multidrug resistance genes. The comparison of antimicrobial resistance phenotypes and genotypes of toxigenic *C. difficile* isolates showed general concordance (Supplementary Figure 1). High resistance to clindamycin and erythromycin was largely consistent with the presence of *ermB* (31 strains). Similarly, tetracycline resistance was associated with the presence of TET genes, including *tetM* (18 strains), *tetA(P)*, and *tetB(P)*. Elevated moxifloxacin MICs were strongly linked to the Thr82Ile mutation in *gyrA* (13 strains). The overall resistance phenotypes showed good agreement with the major resistance determinants. Furthermore, mobile genetic element analysis identified four transposons among the toxigenic *C. difficile* isolates, including Tn*916*, Tn*5251*, Tn*6189*, and Tn*6194*-like. Tn*916*, carrying *tetM*, was the most frequently detected transposon and was identified predominantly in ST81, ST129, and ST37 isolates. Tn*6189*, carrying *ermB*, was detected in several ST81 and ST3 isolates, whereas Tn*5251*, carrying *tetM*, was identified only in the single ST415 isolate. Tn*6194*-like, carrying *ermB*, was detected only in the ST37 isolate.

### Factors associated with CDI

The distribution of CDI differed significantly across departments, especially in the ICU and rehabilitation department (*P* < 0.001, Supplementary Table 2). In univariate analysis, rehabilitation department and ICU were associated with increased odds of CDI. In the exploratory multivariable logistic regression analysis, patients from the rehabilitation department (aOR 5.05, 95% CI 2.04–12.54, *P* < 0.001) and ICU (aOR: 14.66, 95% CI: 4.55–47.28, *P* < 0.001) showed significantly higher odds of CDI than patients from the other departments (Table 2). Age and sex were not significantly associated with CDI (Figure 1B).

**Table 2:** Multivariable analysis for *Clostridioides difficile* infection.

Variable	Unadjusted OR (95% CI)	*P* value	Adjusted OR (95% CI)	*P* value
Age (per 10-year increase)	1.06 (0.94–1.20)	0.311	1.15 (0.97–1.36)	0.109
Male sex	1.32 (0.74–2.34)	0.349	1.32 (0.73–2.39)	0.352
Digestive department	0.83 (0.45–1.52)	0.541	1.62 (0.78–3.38)	0.199
Rehabilitation department	3.57 (1.61–7.92)	0.002	5.05 (2.04–12.54)	< 0.001
Pediatric department	1.40 (0.62–3.17)	0.425	4.69 (1.24–17.64)	0.022
ICU	10.69 (3.65–31.28)	< 0.001	14.66 (4.55–47.28)	< 0.001

## Discussion

*C. difficile* infection continues to represent a considerable challenge for public health and represents a significant burden for global healthcare systems (Rodriguez et al., 2016; Markantonis et al., 2024). In addition to cross-transmission within and even between hospitals, outbreaks have also been reported in healthcare settings (Jia et al., 2016; Shu et al., 2023). Undoubtedly, the persistent transmission potential of *C. difficile* further emphasizes the critical importance of enhancing the focus on the genomic epidemiological surveillance of *C. difficile*. In our study, the isolation rate of toxigenic *C. difficile* was found to be only 3.8%, which was lower than the rates reported by other hospitals (Xu et al., 2017; Lu et al., 2026; Chen et al., 2018). This relatively low isolation rate of toxigenic *C. difficile* may be largely attributable to the characteristics of the hospital itself. The clinical stool samples in this study were collected from a county-level hospital, where both patient volume and case complexity are substantially lower than those in large tertiary-care hospitals. Furthermore, the implementation of routine clinical testing for *C. difficile* had not been instituted, and clinicians lacked adequate awareness and suspicion of CDI when managing patients with diarrhea. Therefore, these factors may have contributed to the underdiagnosis of *C. difficile* infection as well as insufficient submission of diarrheal stool samples for testing.

Despite the relatively low isolation rate of toxigenic *C. difficile* in the present study, a comparison of the isolation rate of toxigenic *C. difficile* strains among different departments revealed variable rates of isolation from different departments, with higher isolation rates of toxigenic *C. difficile* observed in the ICU and rehabilitation department. These results are consistent with other reports showing that the incidence of CDI was highest in the ICU (Chen et al., 2014; Schindler et al., 2013). This phenomenon may be associated with factors such as extensive antibiotic exposure, older age of patients and underlying illness, all of which have been found to be significantly associated with *C. difficile* infection (Young et al., 1986; Ananthakrishnan, 2011; Miles-Jay et al., 2023). Actually, ICU patients in the present study had a relatively advanced mean age of 67 years, and the risk of developing CDI was found to be significantly increased in this department. Similarly, the higher isolation rates of toxigenic *C. difficile* and risk observed in the rehabilitation department were likely indicative of cumulative healthcare exposure, older age and prolonged hospitalization, all of which also have been demonstrated to be significantly associated with *C. difficile* infection (Ananthakrishnan, 2011). These observations underscore the strong association between CDI and healthcare-associated frailty in hospitalized patients.

*C. difficile* demonstrates a high genetic diversity, with molecular epidemiological patterns exhibiting both geographic heterogeneity and shared epidemic lineages across different regions (Miles-Jay et al., 2021; Collins et al., 2020). In the Western countries, the hypervirulent *C. difficile* ribotypes RT027 and RT078 are highly prevalent and have also been isolated in China; however, they have not become the dominant epidemic strains among Chinese populations (Clements et al., 2010; Lv et al., 2019). Meanwhile, although *C. difficile* ST5 (Clade 3) and ST415 (Clade 5) have attracted considerable attention internationally because they are associated with binary toxin-positive lineages, these sequence types accounted for only one isolate each in this study. In contrast, ST2, ST3, ST35, ST37, and ST54 are among the most frequently reported genotypes in mainland China (Wen et al., 2023). Meanwhile, the predominant genotypes may also vary across different geographic regions and healthcare settings in China. In Zhejiang Province, studies from large tertiary hospitals in Ningbo identified ST2, ST3, ST35, and ST54 as the predominant genotypes (Shu et al., 2023; Hu et al., 2024), whereas investigations from Hangzhou reported ST54, ST35, and ST37 as the most prevalent sequence types (Jin et al., 2017; Chen et al., 2014, 2018). In our county-level hospital, the most prevalent types of the toxigenic *C. difficile* were ST2, ST3, ST81, ST35, and ST129. Such regional variation in predominant *C. difficile* genotypes may be associated with the differences in antimicrobial usage, hospital characteristics, patient populations, and local transmission dynamics. In addition, healthcare-associated transmission and clonal expansion within hospitals may further contribute to the predominance of specific sequence types in different regions and healthcare settings (Miles-Jay et al., 2023). Consistent with these observations, the minimum spanning tree analysis in this study revealed small cgSNP differences among several predominant lineages, including ST3, ST35, ST81, and ST129. Closely related isolates with extremely small cgSNP differences were identified not only among patients within the same department, but also between patients from different departments, suggesting the possible intra- and inter-departmental transmission events. Obviously, these findings indicated that both epidemiological characteristics and healthcare-associated transmission dynamics play important roles in shaping the molecular distribution of *C. difficile*. Therefore, continuous genomic surveillance and strengthened infection control measures in hospital settings still remain essential.

Moreover, antimicrobial resistance plays an important role in the pathogenesis and spread of CDI by enabling *C. difficile* to survive under antimicrobial selective pressure due to the relevant antimicrobial resistance genes (Imwattana et al., 2020). Currently, *C. difficile* strains exhibit high levels of resistance to several antibiotics, particularly fluoroquinolones, tetracycline, erythromycin, and clindamycin (Peng et al., 2017). Furthermore, there have even been reports of reduced susceptibility or the resistance to vancomycin and metronidazole, the first-line antibiotics used for the treatment of CDI (Peng et al., 2017). Fortunately, all toxigenic *C. difficile* strains in this study remained susceptible to vancomycin, metronidazole and fidaxomicin. However, the high resistance rates to clindamycin and erythromycin are observed in our study, which are similar to previous reports (Jin et al., 2017; Chen et al., 2018). In our study, most of toxigenic *C. difficile* strains resistant to clindamycin and erythromycin carried *ermB*, whereas tetracycline-resistance isolates predominantly harbored *tetM*. Notably, several of these resistance genes were located on mobile genetic elements, including *tetM* on Tn*916* and Tn*5251*, and *ermB* on Tn*6189* and Tn*6194*-like. These findings suggest that transposon-mediated horizontal gene transfer may be a key factor in spreading antibiotic resistance and play a role in facilitating the dissemination of antimicrobial resistance within the local *C. difficile* population (Jones et al., 2021). Meanwhile, among those strains, all ST81 isolates and most ST3 isolates, the most prevalent types in this study, were resistant to moxifloxacin and detected a *gyrA* (Thr82Ile) mutation. Actually, alterations in the quinolone resistance determining region of GyrA, particularly the Thr82Ile substitution, are recognized as a major mechanism mediating fluoroquinolone resistance in *C. difficile* (Spigaglia, 2016). More importantly, fluoroquinolone resistance resulting from the *gyrA* (Thr82Ile) mutation in the epidemic *C. difficile* RT027/ST1 is thought to have significantly contributed to its rapid expansion and global dissemination during the early 21st century (He et al., 2013). Therefore, molecular epidemiological surveillance of *C. difficile*, particularly for multidrug-resistant strains carrying key resistance determinants, is of great importance for better understanding transmission dynamics and preventing the dissemination of epidemic resistant strains within healthcare settings.

Our research also had several limitations. Firstly, although samples were collected over a 12-month period, the overall sample size remained relatively limited, which may have restricted a more comprehensive characterization of the molecular epidemiological features of *C. difficile* in our hospital. Secondly, despite performing whole genome analysis, environmental samples were not collected during the study period. This prevented the identification of potential transmission routes and sources associated with nosocomial transmission among patients. Furthermore, genomic data obtained from other hospitals within the same geographic region were not included in the analysis. Consequently, we were unable to further elucidate the underlying factors contributing to the geographic heterogeneity and shared epidemic lineages observed in the molecular epidemiological patterns of *C. difficile*, particularly with regard to potential regional transmission events. Furthermore, comprehensive clinical information, including the history of antibiotic therapy, proton pump inhibitor use, underlying comorbidities, and other pertinent information, was not available for all patients. Therefore, these established risk factors could not be comprehensively evaluated in this study.

In conclusion, this study provides insight into the molecular characteristics of *C. difficile* in a county-level hospital in China. Despite the relatively low isolation rate of toxigenic *C. difficile*, several predominant genotypes were identified, and the subsequent whole genomic analysis revealed the genomic evidence compatible with potential nosocomial transmission. Furthermore, high resistance rates to antimicrobial agents and the distribution of key resistance determinants were observed among several predominant genotypes. These findings highlight the important roles of healthcare-associated transmission and antimicrobial selective pressure in shaping the local molecular epidemiological patterns of *C. difficile*. Continuous genomic surveillance and strengthened infection prevention and control strategies remain essential for limiting the dissemination of epidemic and multidrug-resistant *C. difficile* strains in healthcare settings.

## Data Availability

The datasets presented in this study can be found in online repositories. The names of the repository/repositories and accession number(s) can be found below: https://www.ncbi.nlm.nih.gov/, PRJNA1466775.
