# Supplementary material for: Clinical and molecular epidemiology of Clostridioides difficile infection in a county-level hospital in Zhejiang Province, China
Source: Front Microbiol. 2026 Aug 4;17:1882254. doi: 10.3389/fmicb.2026.1882254 (PMC13481431; doi:10.3389/fmicb.2026.1882254)
Supplement: Supplementary file 1 [file Data_Sheet_1.docx]

**Supplementary Table 1.** Age distribution in each department.

| **Department** | **n** | **Mean age** | **Median** | **Min** | **Max** |
| --- | --- | --- | --- | --- | --- |
| Digestive Department | 474 | 53.86 | 57 | 12 | 92 |
| Pediatric department | 140 | 5.19 | 4 | 1 | 13 |
| Outpatient | 111 | 34.8 | 34 | 4 | 79 |
| Oncology department | 87 | 65.32 | 68 | 20 | 88 |
| Orthopedics department | 78 | 64.96 | 66 | 23 | 87 |
| Department of Cardiology | 76 | 69.51 | 74.5 | 16 | 94 |
| Rehabilitation department | 72 | 64.19 | 63 | 13 | 101 |
| Pneumology department | 70 | 68.17 | 71.5 | 13 | 92 |
| General surgery department | 68 | 61.38 | 63 | 15 | 90 |
| Neurology department | 59 | 73.44 | 77 | 31 | 95 |
| Emergency department | 39 | 37.67 | 38 | 1 | 87 |
| Gynecology department | 19 | 47.84 | 46 | 23 | 79 |
| ICU | 18 | 67 | 74.5 | 24 | 86 |
| Others | 4 | 48 | 56.5 | 1 | 78 |

**Notes:** n: number, Min: Minimum age, Max: Maximum age

**Supplementary Table 2.** isolation rate of toxigenic *C. difficile* infection in each department.

| **Department** | **Total n** | **CDI n** | **Isolation rate (%)** | **Crude OR** | **95% CI lower** | **95% CI upper** | ***P* value** |
| --- | --- | --- | --- | --- | --- | --- | --- |
| ICU | 18 | 5 | 27.8 | 10.7 | 3.66 | 31.3 | 0.00 |
| Others | 4 | 1 | 25 | 8.59 | 0.88 | 84.03 | 0.14 |
| Rehabilitation department | 72 | 8 | 11.1 | 3.57 | 1.61 | 7.93 | 0.00 |
| Gynecology department | 19 | 2 | 10.5 | 3.06 | 0.69 | 13.62 | 0.16 |
| Emergency department | 39 | 2 | 5.1 | 1.38 | 0.32 | 5.91 | 0.66 |
| Pediatric department | 140 | 7 | 5 | 1.39 | 0.61 | 3.14 | 0.48 |
| Digestive Department | 474 | 16 | 3.4 | 0.83 | 0.45 | 1.52 | 0.65 |
| Oncology department | 87 | 3 | 3.4 | 0.9 | 0.27 | 2.94 | 1.00 |
| General surgery department | 68 | 2 | 2.9 | 0.76 | 0.18 | 3.18 | 1.00 |
| Orthopedics department | 78 | 2 | 2.6 | 0.65 | 0.16 | 2.73 | 0.76 |
| Neurology department | 59 | 1 | 1.7 | 0.42 | 0.06 | 3.13 | 0.72 |
| Department of Cardiology | 76 | 1 | 1.3 | 0.32 | 0.04 | 2.38 | 0.36 |
| Outpatient | 111 | 0 | 0 | 0.1 | 0.01 | 1.67 | 0.02 |
| Pneumology department | 70 | 0 | 0 | 0.17 | 0.01 | 2.75 | 0.11 |

**Notes:** n: number

**Supplementary Figure 1.** Antimicrobial susceptibility profiles, antimicrobial resistance determinants, and resistance-associated amino acid substitutions of the 50 toxigenic *C. difficile* isolates.
